# Innovation of EUS-guided transmural gallbladder drainage using a novel self-expanding metal stent

**DOI:** 10.1038/s41598-020-67374-y

**Published:** 2020-07-07

**Authors:** Gunn Huh, Jin Ho Choi, Sang Hyub Lee, Woo Hyun Paik, Ji Kon Ryu, Yong-Tae Kim, Seok Jeong, Don Haeng Lee, Gyeong Hwan Kim, Sung Gwon Kang

**Affiliations:** 1 Division of Gastroenterology, Department of Internal Medicine, Liver Research Institute, Seoul National University Hospital, Seoul National University College of Medicine, 101 Daehak-ro, Jongno-gu, Seoul, 110-744 Korea; 2 Department of Internal Medicine, Inha University School of Medicine, Incheon, Korea; 3 S&G Biotech Inc., Yongin, Korea

**Keywords:** Gastroenterology, Gastrointestinal diseases, Biliary tract disease, Pancreatic disease

## Abstract

Endoscopic ultrasonography (EUS)-guided transmural drainage has been accepted as a modality of choice in peripancreatic fluid collection and acute cholecystitis. Each type of stent, including double-pigtail plastic stents, tubular self-expandable metal stents (SEMS), and lumen-apposing metal stents, for these procedure has its own advantages and disadvantages. To overcome their disadvantages, this animal study evaluated the feasibility of a newly designed twisted fully covered SEMS with spiral coiled ends. We performed the EUS-guided cholecystogastrostomy with a newly developed metal stent in eight mini pigs with surgically induced gallbladder distension. This novel stent is a twisted fully covered SEMS with spiral coiled ends, a diameter of 8 mm, and a length of 6 cm. The stent has been maintained for four to seven weeks after EUS-guided cholecystogastrostomy. The primary outcome was the technical success rate, and the secondary outcomes were adverse events, stent dysfunction, stent removability, and fistula formation. The stent was placed successfully between the gallbladder and the stomach in all cases without any adverse event. We observed neither stent migration nor dysfunction during the study period, and all the stents were removed easily as scheduled. We confirmed successful cholecysto-gastric fistula formation at endoscopic and histologic level in all cases. EUS-guided transmural drainage and fistula formation using a new twisted fully covered metal stent with spiral coiled ends was technically feasible without any adverse event in this animal study. Further clinical studies are needed to evaluate its efficacy and safety in real practice.

## Introduction

Endoscopic ultrasonography (EUS)-guided transmural drainage is accepted as a modality of choice when drainage is required in several diseases, such as acute cholecystitis or peripancreatic fluid collection (PFC) including pancreatic pseudocysts, and walled-off necrosis of the pancreas^1–3^. Conventional approaches in these situations are to mount double-pigtail plastic stents (PS) or tubular self-expanding metal stents (SEMS). Placement of a PS is technically difficult even with EUS guidance, involving placement of multiple stents and repeated guidewire access^4^. Recently introduced lumen-apposing metal stents (LAMS) have a relatively large, relatively stiff delivery system, which can be technically demanding for endoscopist unfamiliar with LAMS^5^.

Endoscopists recognized that PSs are easily clogged, prone to secondary infection because of their small caliber, and require multiple stents for effective drainage^6^, whereas tubular SEMS are prone to migrate and leak despite their larger diameter that enables clinical improvement with a single-session procedure^7^. LAMS show improvement in efficacy and are expected to have advantages over other stents in the management of fluid collections with solid debris^3,8–10^. A multi-institutional consensus recommended that LAMS should be the standard modality for PFC^11^. However, several studies reported that severe adverse events such as lethal bleeding and stent migration might occur from LAMS^9,12–15^. In addition, not only short tubular SEMS but also some types of LAMS sometimes migrated to extraluminal space due to accidental deployment in real practice. Therefore, there is still a need to improve existing stents by developing newly designed stents.

The aims of this animal study with a pig model are to evaluate the feasibility, safety, and removability of the newly designed twisted, fully covered SEMS with spiral coiled ends.

## Methods

### Animal model

Eight male mini pigs (*Sus scrofa*) weighing 24–36 kg were used for the experiment. A sample size of eight pigs was determined based on previous animal studies and statistical consideration^7,16^. With an adverse event rate of 20%, an estimation based on clinical studies on EUS-guided transmural drainage^17–19^, eight pigs were required to observe at least one adverse incident at an 80% confidence interval^20^. The animals were housed at an animal research facility (KNOTUS Co., Ltd., Guri, Gyeonggi Province). Five days before the procedure, the common bile duct (CBD) was ligated with surgical suture to provoke an enlarged gallbladder (GB). We obtained institutional review-board approval from the local animal ethics committee (The Institutional Animal Care and Use Committee at the KNOTUS Co. Ltd, Gyeonggi-do, Korea, IRB No. IACUC-18-KE-459). This study was written in adherence to the ARRIVE guideline^21^.

### Newly designed twisted, fully covered, self-expandable metal stent with spiral coiled ends for endoscopic ultrasonography-guided transmural drainage

**Figure 1:**
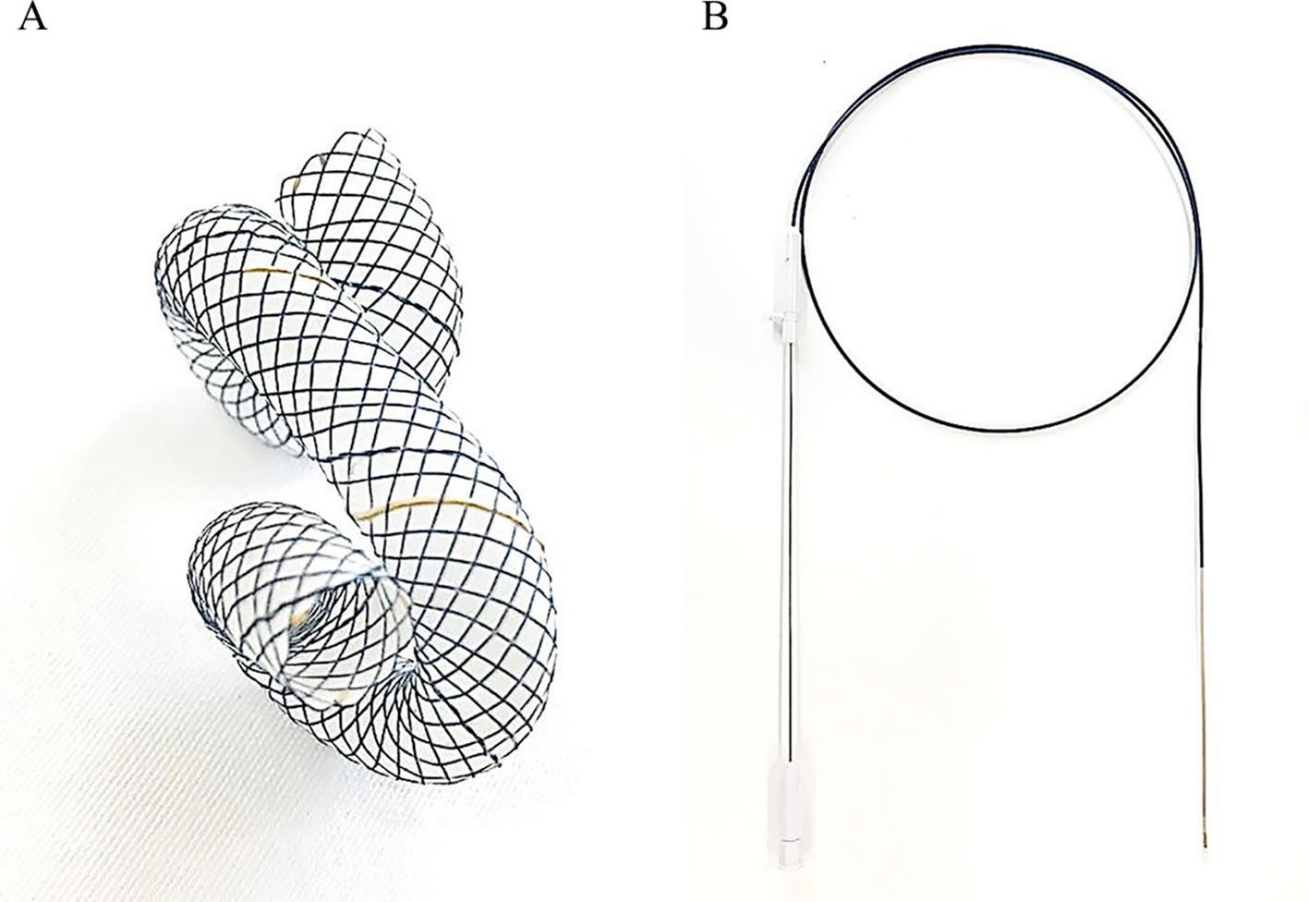
The Tornado stent: a novel fully covered self-expandable metal stent with spiral coiled ends. (**A**) In vitro appearance of Tornado stent. (**B**). A 8F conventional delivery system which includes radio-opaque markers at both ends and the center of delivering catheter.

Tornado stent (S&G Biotech, Inc., Yongin, South Korea) is a braided, twisted SEMS with spiral coiled ends, which is made of nitinol wire and is fully covered with silicone (Fig. 1A). Both pigtail ends are spiral coiled in order to minimize the risk of bidirectional migrations. The diameter of the stent is 8 mm. The length of the stent is 22 cm when constrained in the delivery catheter, 14 cm when the outer sheath is removed during the deployment process, and 6 cm in coiled form when fully deployed. In ex vivo experiment using a water tank, set to maintain a constant temperature of 37 degrees Celsius, the stent shortening rate of the central straight part of the stent was 25% in 48 h. Radio-opaque markers are located at both ends and at the central straight part of the stent.

The stent is delivered through an 8 Fr catheter, which is compatible with the working channel of an echoendoscope (Fig. 1B; Video 1). The delivering catheter is tipped with a radiopaque olive and made of PVC. The outer diameter of 8Fr is similar to that of delivering catheter used in commercially available fully covered SEMS and smaller than that of LAMS, which ranges from 9Fr to 10.8Fr. The delivering catheter also has radio-opaque markers at both ends and at the center of the delivering catheter, which helps to insert the stent in the exact position under fluoroscopic guidance. The stent deployment process is quite similar to that in most conventional biliary SEMS. After the delivering catheter is advanced into the desired position, the outer restraining sheath is incrementally withdrawn by an assistant to release the stent. The endoscopist needs to apply a graded withdrawal of the delivering catheter while constantly monitoring fluoroscopy to locate the central radiopaque marker of the stent at the center of walls. And at this moment, the endoscopist should make sure through the endoscopic view that the stent is being released at the proper location. After the complete withdrawal of the outer sheath, the endoscopist pulls of the core catheter, and the both ends of the stents are coiled 360 degrees.

### EUS-guided cholecystogastrostomy

**Figure 2:**
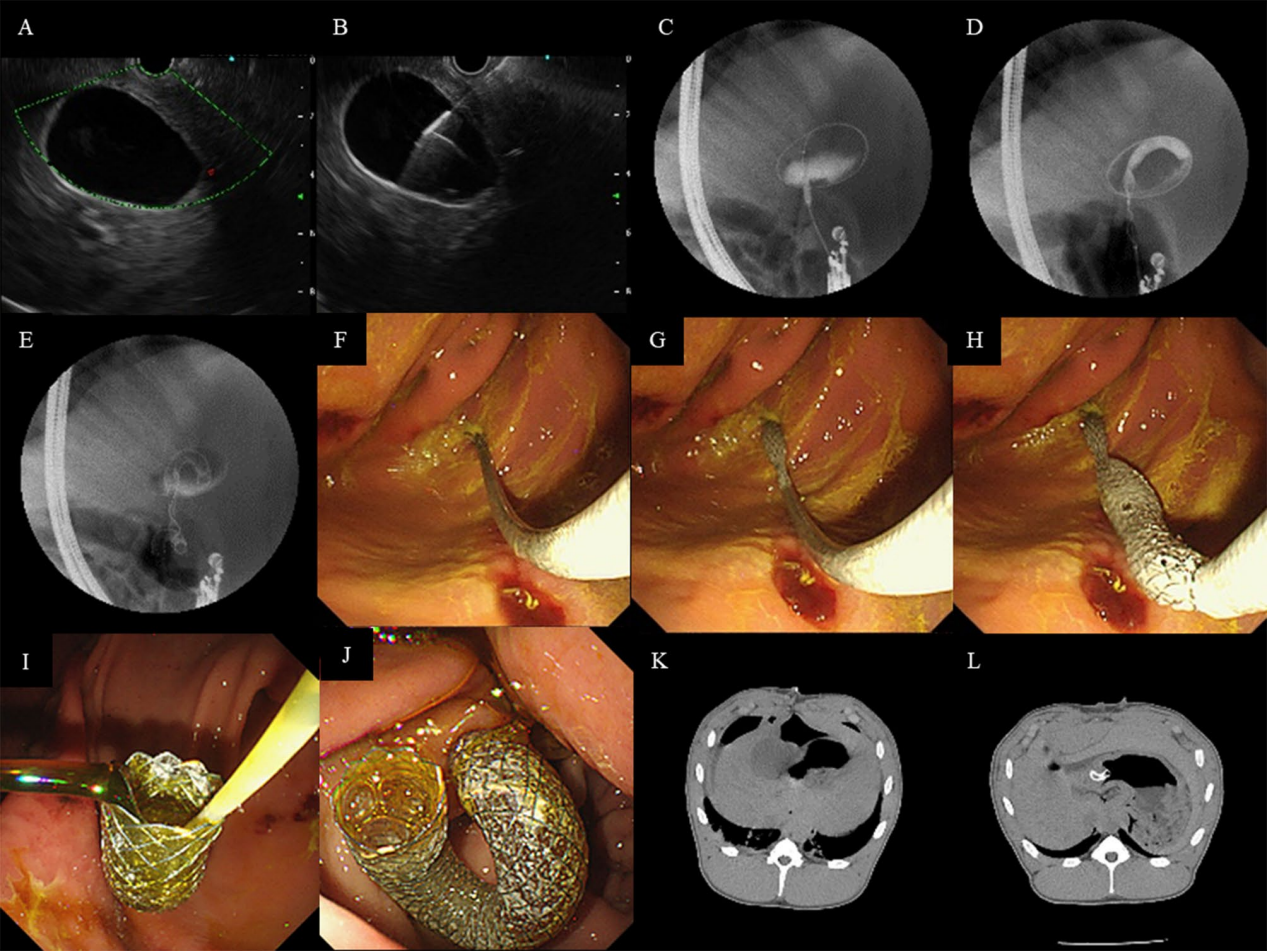
EUS-guided cholecysto-gastrostomy using a novel stent. (**A**) Assessment of regional vascularity with color Doppler, (**B**) puncture of GB by a 19G needle under EUS guidance. (**C**) Balloon dilatation of puncture site. (**D**–**J**) Stent deployment under fluoroscopic, EUS and endoscopic guidance. (**J**) Endoscopic view of the fully deployed stent. (**K**) Preoperative CT. (**L**) CT performed at post-operative 2 weeks showing the stent located between GB and stomach.

All EUS procedures were performed by a single expert in interventional endosonography (S.H.Lee). We imposed 48 h of fasting to induce the distension of the GB before the procedure. Endotracheal intubation was done under general anesthesia. The GB was assessed by EUS processor (EU-ME2 Premier Plus; Olympus, Tokyo, Japan) and a linear array echoendoscope (UF-260C; Olympus). The EUS probe was advanced into the anterior wall of the stomach and we used color Doppler to identify regional vasculature. Since the GB was better visualized from the stomach than the duodenum, we used transgastric approach as in previous animal models^7,16^. The GB was punctured with a 19-gauge needle (EZShot 3 Plus; Olympus) under direct sonographic visualization from the stomach. The site of the least combined wall thickness of the GB and the stomach, which was less than 20 mm in all cases, was selected as a puncture site (Fig. 2B). After confirming that yellowish bile was regurgitated into a syringe, we did cholecystography to ensure that the GB was punctured correctly without leakage. We inserted a 0.025-inch guidewire (EEGW35450; S&G Biotech, Inc.) through the access needle and coiled it about three turns inside the GB. After removing the access needle, we did a further puncture with a cystotome (Optimos OCT1906; Taewoong Medical Co, Ltd, Ilsan, South Korea). An additional tract dilatation with a 4-mm or 6-mm balloon catheter (Hurricane RX Biliary balloon; Boston Scientific, Natick, Mass) for 30 s was performed in 4 pigs, where there was resistance during the advancement of the 8Fr delivering catheter. Finally, we inserted the delivering catheter over the guidewire, and deployed the stent (Tornado stent; S&G Biotech, Inc.) under the guidance of fluoroscopy, EUS and endoscopy.

### Clinical outcomes

The primary outcome was the rate of technical success defined as the successful placement of a stent between the GB and the stomach. The secondary outcomes included adverse events, stent dysfunction because of migration or occlusion, stent removability, and the presence of matured cholecysto-gastric fistula in radiologic and histopathologic exams. Adverse event refers to unexpected incidents that lead to harm, or endanger the well-being of animals and humans at a research facility as defined by the National Institutes of Health-Office of Laboratory Animal Welfare (NIH-OLAW)^22^. Removability was defined as the ability to remove the stent by endoscopy without stent related adverse events after predefined follow-up periods.

### Follow-up, stent removal, and necropsy

We did follow-up computed tomography (CT) two weeks after stent insertion. Follow-up periods were 28, 35, 42, and 49 days for each pair of two pigs. The different follow-up periods were set to evaluate the minimum stent maintenance period needed for fistula tract formation and maximum period to allow the smooth endoscopic stent removal. After the 28 to 49 days of follow-up, we used gastroscopy to evaluate the patency and the removability of a stent. We removed stents with a snare (POL1-D1-30-23-220-OL; Medwork, Höchstadt, Germany) and inserted a slim gastroscope with 5.4-mm distal-end diameter (GIF-XP260NS; Olympus) through the cholecystogastric tract to evaluate the lumen of GB. Contrast was injected under fluoroscopy to check the formation of a fistula tract without any leakage. We performed necropsy one day after stent removal to evaluate the maturation of cholecystogastric fistula tract.

## Results

All pigs showed normal behavior without signs of infection or adverse events during the follow-up period. Study outcomes of the eight pigs are presented in Table 1. The stents were deployed successfully without any technical difficulty in any of the eight pigs (Fig. 2; Video 2). There was no adverse event during the procedure nor any early or late adverse event such as bleeding or perforation during the follow-up period of 28–49 days (median, 38.5 days).

**Table 1:** Outcome of EUS-guided cholecysto-gastrostomy.

	Technical success	Procedure time^a^ (minutes)	Maintenance period (days)	Patent stent	Removal success	Adverse event		Fistula tract
						Early	Late	
Pig 1	Y	42	28	Y	Y	N	N	Y
Pig 2	Y	31	28	Y	Y	N	N	Y
Pig 3	Y	21	35	Y	Y	N	N	Y
Pig 4	Y	24	35	Y	Y	N	N	Y
Pig 5	Y	22	42	Y	Y	N	N	Y
Pig 6	Y	19	42	Y	Y	N	N	Y
Pig 7	Y	13	49	Y	Y	N	N	Y
Pig 8	Y	15	49	Y	Y	N	N	Y

*EUS* endoscopic ultrasonography.

^a^The time interval from the visualization of gallbladder by EUS to the completion of deployment of the stent.

**Figure 3:**
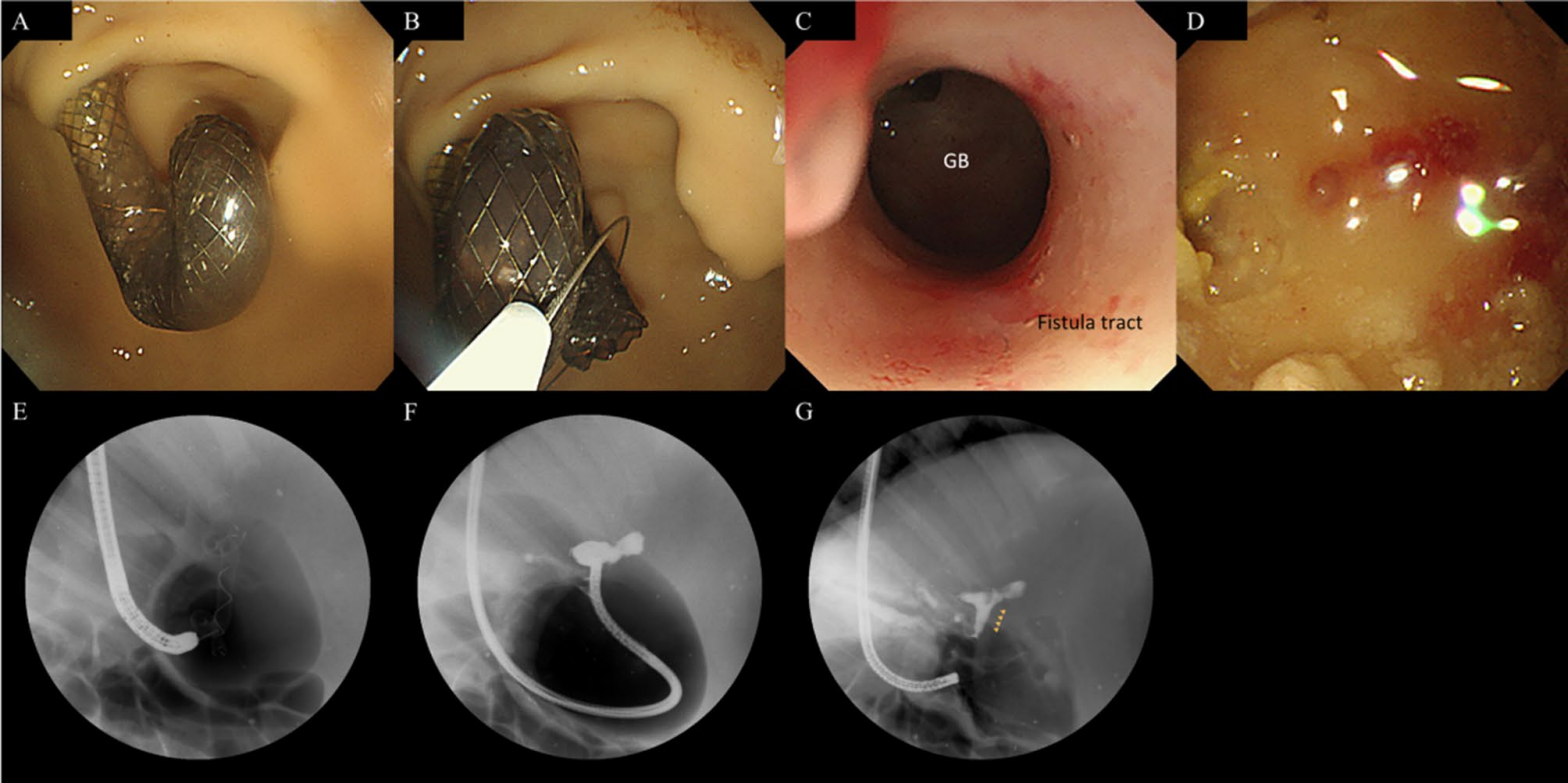
Follow-up endoscopy performed at 42 days after EUS-guided cholecysto-gastrostomy. (**A**, **B**) Endoscopic view showing removal of a stent by a snare. (**C**, **D**) Endoscopic view of lumen of gallbladder through the fistula tract by slim gastroscope. (**E**) Radiograph showing removal of a stent by a snare. (**F**, **G**) Contrast injection revealing a patent cholecysto-gastric fistula tract without any leakage (arrow: fistula tract).

We did the follow-up endoscopy exams as scheduled in all pigs and found patent and stable stents that were well maintained in shape without any evidence of occlusion or migration (Fig. 3A). Stents were removed easily from all pigs (Fig. 3B; Video 3) without any adverse event.

**Figure 4:**
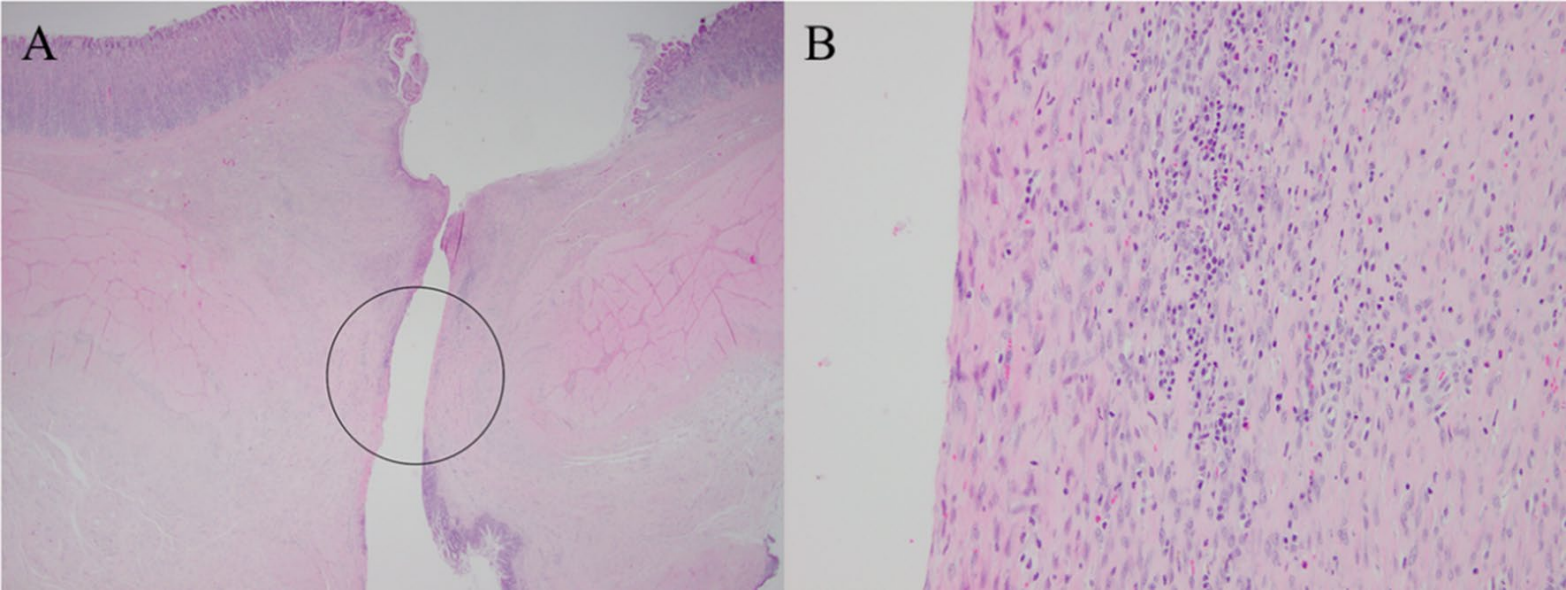
Microscopic findings of necropsy showing the remodeling of tissue around cholecysto-gastric fistula by wound healing. (**A**) Loss of muscle tissue around the fistula tract (hematoxylin and eosin stain, × 12.5). (**B**) Fibroblast hyperplasia with chronic inflammatory cell infiltration (hematoxylin and eosin stain, × 200).

A slim gastroscope could be inserted through the cholecystogastric fistula tract to observe the lumen of the GB (Fig. 3C,D). Cholecystography revealed a patent cholecystogastric tract with no leakage (Fig. 3E–G). The necropsy revealed the well-matured cholecystogastric fistula tract with remodeling of tissue around the fistula by wound healing. On the histopathologic exam, we observed loss of muscle tissue and fibroblast hyperplasia with chronic inflammatory cell infiltration around the fistula tract. (Fig. 4).

## Discussion

We conducted this animal study with a pig model to evaluate the feasibility, safety, and removability of a newly developed twisted, fully covered, self-expandable metal stent with spiral coiled ends (Tornado stent; S&G Biotech, Inc., Yongin, South Korea), which is expected to be easily removable after formation of the fistula while ensuring sufficient anchoring capability. Tornado stent is designed to focus on improving tubular SEMS by combining advantages of double-pigtail PS and tubular SEMS to achieve properties such as firm anchoring, easy removability, sufficient stent caliber, and guarantee of good stent patency.

In this study, we found that Tornado stent was placed successfully without technical difficulties or adverse events and removed easily while creating a firm fistula tract in all eight pigs. The duration of 35–42 days is a proper maintenance period for maturation of the fistula tract on the basis of this study. There has been an ongoing attempt to use SEMS for EUS-guided transmural gallbladder or PFC drainage instead of PS, because of technical difficulty and small diameter of PS^4^. Despite the relatively easy procedure using tubular SEMS, higher incidences of leakage or stent migration into peritoneal cavity had precluded a wider use of SEMS, until recently successful results with LAMS were reported. These adverse events of tubular SEMS are mainly due to the weak anchoring force and occur more frequently in GB drainage because of insufficient attachment between GB and stomach or duodenum. For EUS-guided cholecystogastrostomy, the technical or clinical success rates between PS, tubular SEMS, and LAMS were similar, but adverse events such as leakage or stent migration were slightly higher in SEMS with 4.8–28.5%^17–19^, whereas those of LAMS are slightly lower at 5.8–13%^1,23–25^. In cases of EUS-guided drainage and debridement for PFC, tubular SEMS and LAMS were superior to PS in terms of overall treatment efficacy, and the number of procedures required for resolution was lowest in LAMS^9^. The emerging concerns for LAMS that need improvement in the future are the recently reported lethal adverse event including bleeding or buried stent syndrome and the technical difficulty of rescue procedure for misdisplacement^7,14,15,26^.

Tornado stent overcomes shortcomings of stents in use as follows. First, its relatively flexible spiral coiled ends could minimize the risk of serious adverse events like bleeding or buried stents. In our study, there was no adverse event during the stent maintenance period, and stents were removed successfully in all cases without any resistance during the removal process. According to the previous study, large flange of LAMS has the potential risk of blocking the pyloric ring in EUS-guided cholecystostomy with transgastric approach^27^. The risk of mechanical complications like this may be lower since the spiral coiled ends of both sides have a fixation effect without a flange in Tornado stent. Second, spiral coiled ends would lower the risk of migration compared with tubular SEMS. Since the coiled ends are flexible, the force from axial direction may decrease, which leads to the prevention of stent migration. In this study, there was no stent migration during the stent maintenance period and follow-up endoscopy exams showed stable stents in all cases. Third, it would lower the infection and necessity of revision rates compared with PS by inhibiting ascending infection with its larger caliber and spiral coiled ends. The stent patency was maintained well in this study, and there was no evidence of infection during the follow-up period. Last, we confirmed a successfully formed fistula at the endoscopic and histologic levels in all cases without any perforation or leakage.

Tornado stent has several drawbacks as well. First, direct endoscopic procedure through the stent or the fistula tract is mostly limited. The fistulae that were formed in this animal study seem to pass only with the thin-caliber nasal scope with 5.4 mm width, so it would not be suitable for a situation that requires further procedures with endoscopic devices, such as direct necrosectomy for walled-off necrosis. However, we think a simple endoscopic procedure, such as electrohydraulic lithotripsy for a remnant stone, might be possible. Second, the stent patency may not be as good as that of conventional tubular SEMS or LAMS because of its smaller caliber. Therefore, the clinical features in the management with Tornado stent might be similar to those with PS except for the necessity of multiple procedural sessions for fistula formation in PS. Third, multiple steps for stent insertion were required with this prototype of the stent because the stent delivery system does not include electrocautery device. Cautery-enhanced catheter in LAMS enables simplified procedure with minimal over-the-wire exchanges of instruments during which complications such as bile leakage can occur. We are planning to develop stent delivery system with electrocautery device after conducting a pilot study of this stent in patients with acute cholecystitis or pancreatic fluid collection.

In conclusion, the feasibility, safety, and removability of EUS-guided transmural gallbladder drainage using a newly designed SEMS (Tornado stent) were proved through this animal study. We expect that the results from the pig model of gallbladder distension would contribute to validate the safety and the efficacy in drainage for PFC as well as in the gallbladder. Further studies on the application of this stent in humans and comparative clinical studies with existing stents will be needed to prove its feasibility, safety and efficacy in real practice. Tornado stent is expected to be a good option for endoscopists who are unfamiliar with LAMS and prefer to avoid the complications in LAMS in the near future.

## Supplementary information

Supplementary Video 1.

Supplementary Video 2.

Supplementary Video 3.
